# First-line cetuximab versus bevacizumab for *RAS* and *BRAF* wild-type metastatic colorectal cancer: a systematic review and meta-analysis

**DOI:** 10.1186/s12885-019-5481-z

**Published:** 2019-03-28

**Authors:** Bobo Zheng, Xin Wang, Mingtian Wei, Quan Wang, Jiang Li, Liang Bi, Xiangbing Deng, Ziqiang Wang

**Affiliations:** 1 Department of Gastrointestinal Surgery, West China Hospital, Sichuan University, Chengdu, 610041 China; 2 Department of Gastroenterology, First Affiliated Hospital of Xi’an Jiaotong University, Xi’an, 710061 Shaanxi Province China; 3 Digestive disease hospital, Xijing Hospital, The Fourth Military Medical University, Xi’an, 710032 Shaanxi China; 4 National Cancer Center/Cancer Hospital, Chinese Academy of Medical Sciences and Peking Union Medical College, Beijing, 100021 China

**Keywords:** First line, Cetuximab, Bevacizumab, Wild type, Metastatic colorectal cancer

## Abstract

**Background:**

A first-line biologic treatment for metastatic colorectal cancer (mCRC) is still controversial. We, therefore, performed a meta-analysis to determine the efficacy of first-line cetuximab versus bevacizumab for RAS and BRAF wild-type mCRC.

**Methods:**

In March 2018, an electronic search of the following biomedical databases was performed: PubMed, EMBASE, Cochrane Library, ClinicalTrials.gov and Web of Knowledge. Randomized controlled trials (RCTs) and prospective or observational cohort studies (OCSs) were included. Subgroup analyses of all RCTs were performed in all outcomes. All statistical analyses were performed using RevMan software 5.3.

**Results:**

Two RCTs and three OCSs, involving a total 2576 patients, were included. The meta-analysis reported that cetuximab was associated with a longer overall survival (OS) [HR 0.89, 95% CI (0.81–0.98); *p* = 0.02], a higher ORR [RR 1.11, 95% CI (1.03–1.19); *p* = 0.006], higher complete response [RR 3.21, 95% CI (1.27–8.12); *p* = 0.01] and a greater median depth of response than bevacizumab. However, no significant difference was observed between cetuximab and bevacizumab groups for PFS, DCR, partial response, progressive disease, curative intent metastasectomy, EORR and incidence of grade 3 or higher adverse events. In the subgroup meta-analyses of the RCTs, inconsistent results compared to the main analysis, however, were found, in the ORR, DCR and curative intent metastasectomy.

**Conclusions:**

The current evidence indicates that compared to bevacizumab treatment, cetuximab provides a clinically relevant effect in first-line treatment against mCRC, at the cost of having lower stable disease.

## Background

Colorectal cancer (CRC) is the third most common cause of cancer-related morbidity worldwide [1], with a 14% five-year survival rate in patients with an initial diagnosis of metastatic colorectal cancer (mCRC) [2]. Approximately one-quarter of CRC patients will present with synchronous metastatic cancer, and 40–60% CRC will develop metachronous disease eventually [3, 4].

With the increasing use of targeted therapies, including epidermal growth factor receptor (EGFR) and anti-vascular endothelial growth factor (VEGF) antibody, the median overall survival (OS) of mCRC has been raised to approximately 30 months over the last 10 years [5, 6]. Cetuximab, a chimeric (human–mouse) monoclonal antibody targeting EGFR, can inhibit cancer cell growth and induce apoptosis [7, 8]. As is well known, EGFR can activate numerous intracellular signaling pathways that promote cancer cell proliferation and survival via stimulating transcription of many genes involved in these pathways [9]. Furthermore, studies several trials have shown clinical efficacy of cetuximab in *KRAS* wild-type mCRC [10, 11].

Bevacizumab is a recombinant humanized monoclonal antibody that targets VEGF and inhibits tumor-driven angiogenesis. The AVF2107 trial proved that irinotecan plus bevacizumab could raise the median OS from 15.6 to 20.3 months [hazard ratio (HR) 0.66, *p* = 0.001] [12]. Consequently. in 2004, bevacizumab was accepted as a first-line treatment for mCRC by the United States Food and Drug Administration. A large retrospective cohort study of 1739 patients with mCRC reported that bevacizumab was associated with higher OS, progression-free survival (PFS) and metastasectomy rates of first-line treatment of mCRC [13].

The FIRE-3 trial, which included 592 patients with *KRAS* exon 2 wild-type mCRC, aimed to compare cetuximab plus FOLFIRI or bevacizumab plus FOLFIRI and reported a significantly longer median OS in the cetuximab group (HR 0.77, 95% confidence interval (CI) 0.62–0.96; *p* = 0.017) [5]. However, there were no differences in the PFSs and objective responses, FIRE-3 was formally negative trial and failed the primary end-point (ORR) [5]. It is difficult to understand why cetuximab was not associated with a significantly enhanced proportion of objective responses but presented an improved long-term median OS. A recent RCT produced a paradoxical result, with no difference found in the OS between cetuximab and bevacizumab groups, for mCRC patients, the CALGB 80405 was also formally negative trials and failed the primary end-point (OS) [6]. Thus, the optimal first-line targeted therapy is contradictory for patients with *RAS* and *BRAF* wild-type mCRC. We, therefore, performed a systematic review and meta-analysis to determine the relative efficacy, including long-term survival outcomes and response rates, of first-line treatment with cetuximab versus bevacizumab.

## Methods

### Data sources and searches

The preferred reporting items for PRISMA guidelines [14] were followed in this systematic review. In March 2018, an electronic search of the following biomedical databases was performed: PubMed, EMBASE, Cochrane Library, ClinicalTrials.gov and Web of Knowledge Conference Proceedings Citation Index—Science (CPCI-S). Abstracts and presentations published by the American Society of Clinical Oncology (ASCO; www.asco.org) and the European Society for Medical Oncology (ESMO; www.esmo.org) were also reviewed. The following search terms were used: “metastatic colorectal cancer”, “targeted agent or targeted therapy”, “epidermal growth factor receptor inhibitor or EGFR inhibitor or cetuximab”, “vascular endothelial growth factor inhibitor or VEGF inhibitor or bevacizumab” and “first line”.

### Study selection criteria

Eligible studies were required to meet the following inclusion criteria: RCTs, prospective or observational cohort studies (OCSs) that evaluated the efficacy and safety of first-line cetuximab versus bevacizumab for *RAS* and *BRA*F wild-type mCRC. The exclusion criteria were as follows: case report, reviews, clinical trial registrations with no result, and cohort studies with lines of therapy other than first and less than eight on the Newcastle–Ottawa Scale (NOS) of nonrandomized cohort trials. The most informative articles are included in the present analysis when more than one paper repetitively reported the same study.

### Outcomes and data extraction

The primary endpoints were OS and PFS. Secondary endpoints were as follows: the objective response rate (ORR), disease control rate (DCR), complete response, partial response, stable disease, progressive disease, early objective response rate (EORR, the percentage of patients achieving 20% or more reduction in the sum of diameters of target lesions [per the Response Evaluation Criteria in Solid Tumors (RECIST) guideline] at the first tumor assessment after baseline), hematologic and nonhematologic adverse events (grade ≥ 3), curative intent in secondary surgery for metastases and depth of response (the relative maximum change in tumor size as a percentage compared to the baseline) [15].

Two independent authors extracted the data using a pre-designed data extraction Microsoft Excel form. If these two reviewers could not reach a consensus, all disagreements were resolved by discussion with a third reviewer. An open assessment of the RCTs was performed using the Jadad scale [16], and The Newcastle-Ottawa Scale [17] was used to assess nonrandomized cohort trials. In addition, all outcomes were graded according to the Grading of Recommendations Assessment Development and Evaluation (GRADE) system [18].

### Statistical analysis

For survival outcomes, the HR was used to pool analyses. For dichotomous clinical outcomes, we expressed treatment effects as relative risks (RR) and 95%CI. I^2^statistics were performed to assess the heterogeneity between studies [19]. A fixed-effect model was used to pool analyses. If statistical heterogeneity (> 50%) was found, a random-effect model was employed, generating a more conservative estimate. Subgroup analyses of all RCTs were performed in all outcomes. All statistical analyses were performed using RevMan software (version 5.3; Cochrane Collaboration), and a two-sided *p* < 0.05 was considered statistically significant.

## Results

### Literature search and selection

**Fig. 1:**
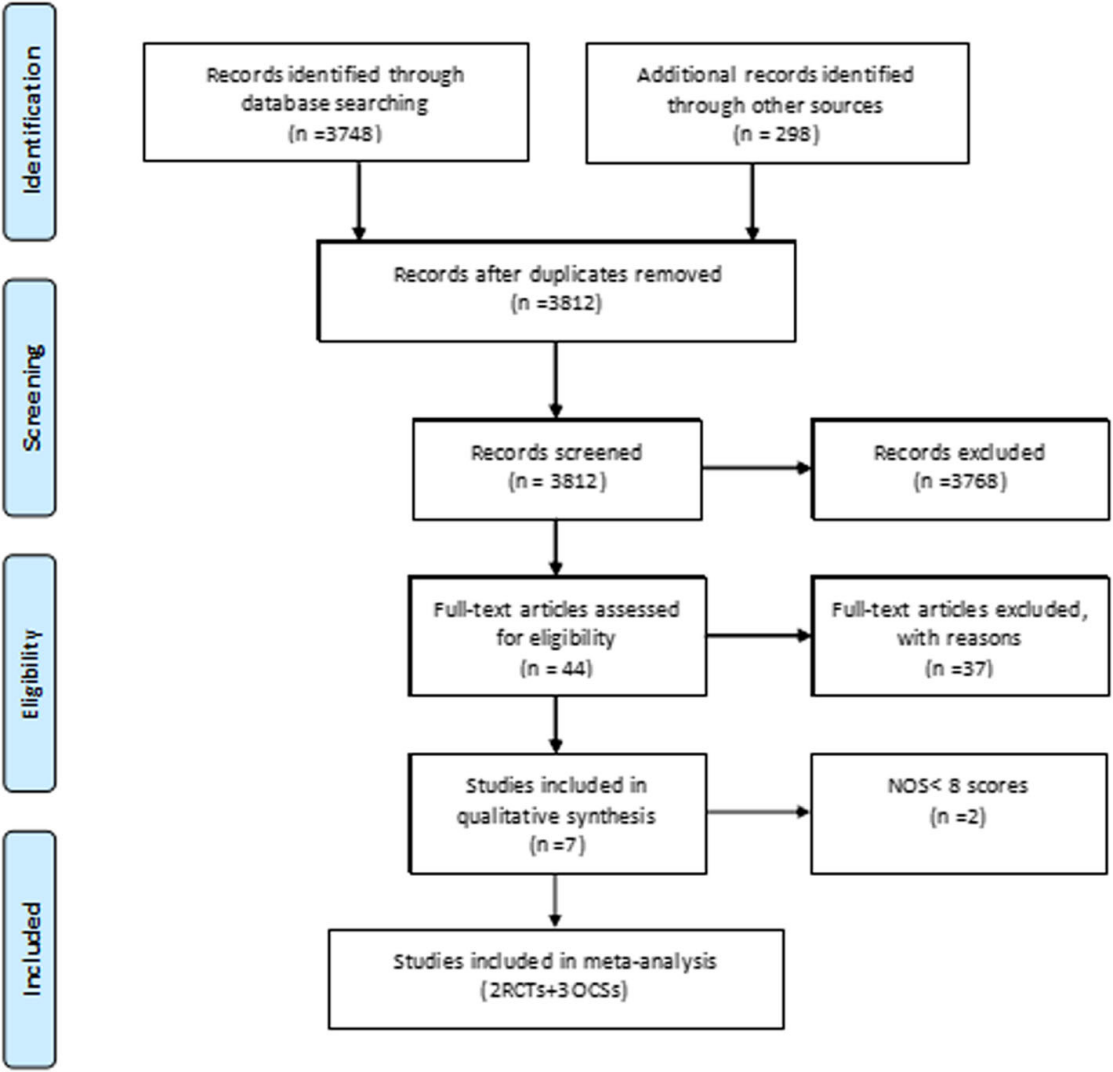
PRISMA 2009 flow diagram

The combined search identified 3812 articles, of which 3768 were excluded, based on the title and abstract evaluation. For the full-text information evaluation, we reviewed the remaining 44 studies. Our exclusion criteria excluded 37 studies. Finally, two RCTs and five OCSs were evaluated. Two OCSs were removed, due to less than eight NOS score [20, 21]. Overall, the meta-analysis included three RCTs [5, 6, 22] (two references [5, 22] involved an RCT) and three OCSs [23–25], comparing first-line cetuximab versus bevacizumab for *RAS* and *BRAF* wild-type mCRC (Fig. 1).

### Study characteristics

The five selected trials were published from Sep 2014 through to Oct 2016 and included patients who received targeted therapies from Apr 2005 through to Dec 2013. Background chemotherapy comprised mFOLFOXIRI [modified irinotecan, oxaliplatin (OXA), 5-fluorouracil (5-FU) and folinate (FOLFOXIRI)], mFOLFOX6 [modified 5-FU, leucovorin (LV) and OXA], FOLFIRI (5-FU, folinate and irinotecan), FOLFOX (5-FU, OXA and LV) or XELOX (capecitabine plus OXA). Overall, these trials analyzed a total 2576 patients receiving targeted therapy. The cetuximab group included 1185 patients, and 758 (63.97%) were male. The bevacizumab group included 1391 patients, and 867 (62.33%) were male. The study quality was rated as high for five studies (Table 1). The baseline characteristics of the five trials are listed in Tables 1 and 2.

**Table 1:** Characteristics of Included Studies

Study	Type	Time	Background therapy, lines of treatment	Outcomes	Followup (media, months)	Jadad/NOS
Venook AP 2017 [6]	RCT	Nov 2005-Mar 2012	mFOLFOX6 or FOLFIRI	OS, PFS and ORR, complete or partial response, 60-day mortality, arterial thrombotic events	47.4	Jadad 5
FIRE-3 [5, 24]	RCT	Mar 2007-Sep 2012	FOLFIRI	objective response, OS, PFS, depth of response, secondary resection of liver metastases with curative intent, early ORR, time to response	40.3	Jadad 5
Houts AC 2017 [26]	OCS	Apr 2005 and Mar 2012	FOLFIRI or FOLFOX	OS, PFS	–	NOS 8
Bai L 2016 [27]	OCS	Jan 2009 - Dec 2013	mFOLFOX-6, XELOX, or FOLFIRI	OS, PFS, ORR, DCR	–	NOS 8
Yang YH 2014 [28]	OCS	Apr 2005-Mar 2012	FOLFIRI or FOLFOX	OS, PFS, ORR, DCR	–	NOS 8

*RCT* Randomized Clinical Trial, *OCS* observational cohort study, *PFR* progression-free rate, *PFS* Progression free survival, *ORR* objective response rate, *OS* overall survival, *DCR* isease control rate

**Table 2:** Characteristics of Included Studies

Study	TN	Age(C/B,yeas) (Median, range)	Number (C/B)	Male(C/B)	SM(C/B)	Primary Tumor Site(C/B)	Metastatic cancer in one Site(C/B)
Venook AP 2017	1137	59.2 (20.8–89.5)/59.0 (21.8–85.0)	578/559	349/348	447/445	Ri:138,L:355,T:31,T:1,Un:53/Ri:142,L:34,T:31,T:0,Un:52	187/165(liver only)
FIRE-3	592	<65y:158/160;>65y:139/135	297/295	214/196	Un	Co:168,Re:115/Co:177,Re:106	119/123
Houts AC 2017	400	61.8 ± 12.65/61.7 ± 11.77	146/254	93/140	73/167	Ri:43,L:92,Un:11/Ri:79,L:162,Un:13	Un
2016 Bai L	289	55 (21–83)/50 (24–79)	101/188	68/120	Un	Co:65,Re:36/ Co:116,Re:70	64/106
2014 Yang YH	158	<60Y:38/43; >60Y:25/52	63/95	34/63	Un	Co:49,Re:14/ Co:72,Re:23	Un

*TN* total number, *SM* Synchronous Metastases, *C* cetuximab, *B* bevacizumab, *Ri* Right, *L* left, *Re* rectum, *T* Transverse, *M* Multiple, *Un* unknown, *Co* colon

### Primary outcomes

**Fig. 2:**
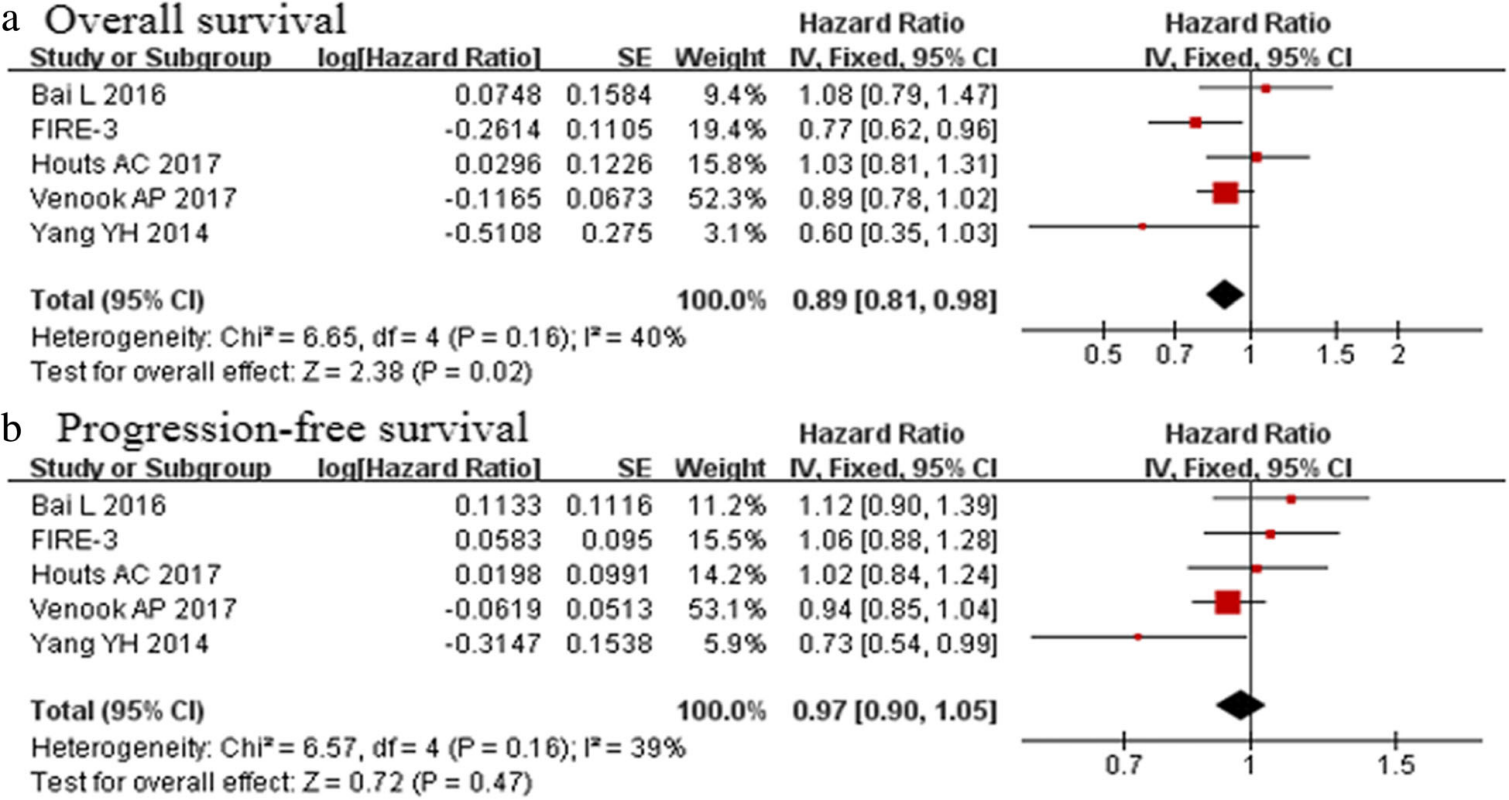
Overall survival and progression-free survival **a** The median OS was better in the cetuximab group than the bevacizumab group; **b** the median PFS did not differ significantly between patients treated with cetuximab and bevacizumab)

Five studies reported the median OS and PFS (Table 3). For the cetuximab group, the median OS ranged from 28.3 to 37.8 months, whereas the bevacizumab group displayed a median OS ranging between 25 and 31.04 months. The median OS was better in the cetuximab group than the bevacizumab group [HR 0.89, 95% CI (0.81–0.98); *p* = 0.02, Fig. 2a, Table 4]. For the cetuximab group, the median PFS was in the range 8.7–12.4 months, whereas the median PFS varied between 8.7 and 10.82 months for the bevacizumab group. The meta-analysis showed that the median PFS did not differ significantly between patients treated with cetuximab and bevacizumab [HR 0.97, 95% CI (0.90–1.05); *p* = 0.47, Fig. 2b, Table 3].

**Table 3:** Overall survival and progression free survival in all included trials

Study(C/B)	Median OS (Months)		Median PFS (Months)	
	C	B	C	B
Venook AP 2017	30	29	10.5	10.6
FIRE-32014	28.7	25	10	10.3
Houts AC 2017	30.64	31.04	10.19	10.82
Bai L 2016	28.3	27.7	8.7	10.6
2014 Yang YH	37.8	30.5	12.4	8.7

**Table 4:** GRADE score for outcomes

Outcomes	No of Participants (studies) Follow up	Quality of the evidence (GRADE)	Relative effect (95% CI)	Anticipated absolute effects	
				Risk with Control	Risk difference with Objective Response Rate (95% CI)
Objective Response Rate	2143 (4 studies)	⊕ ⊕ ⊕⊝MODERATE	RR 1.11 (1.03 to 1.19)	Study population	
				534 ORR per 100	59 more per 1000 (from 16 more to 101 more)
				Moderate	
				513 ORR per 1000	56 more per 1000 (from 15 more to 97 more)
Disease Control Rate	1006 (3 studies)	⊕ ⊕ ⊕⊝MODERATE	RR 0.95 (0.90 to 1.00)	Study population	
				872 DCR per 1000	44 fewer per 1000 (from 87 fewer to 0 more)
				Moderate	
				868 DCR per 1000	43 fewer per 1000 (from 87 fewer to 0 more)
Complete response	1006 (3 studies)	⊕ ⊕ ⊕⊝MODERATE	RR 3.21 (1.27 to 8.12)	Study population	
				11 CR per 1000	24 more per 1000 (from 3 more to 77 more)
				Moderate	
				14 CR per 1000	31 more per 1000 (from 4 more to 100 more)
Partial response	1006 (3 studies)	⊕ ⊕ ⊕⊝MODERATE	RR 1.10 (0.98 to 1.23)	Study population	
				505 PR per 1000	50 more per 1000 (from 10 fewer to 116 more)
				Moderate	
				458 PR per 1000	46 more per 1000 (from 9 fewer to 105 more)
Stable disease	1006 (3 studies)	⊕ ⊕ ⊕⊝MODERATE	RR 0.66 (0.54 to 0.81)	Study population	
				357 per 1000	121 fewer per 1000 (from 68 fewer to 164 fewer)
				Moderate	
				431 per 1000	147 fewer per 1000 (from 82 fewer to 198 fewer)
Progressive disease	1006 (3 studies)	⊕ ⊕ ⊕⊝MODERATE			
			RR 1.31 (0.84 to 2.05)	65 PD per 1000	20 more per 1000 (from 10 fewer to 68 more)
				Moderate	
				69 PD per 1000	21 more per 1000 (from 11 fewer to 72 more)
Early objective response rate	330 (1 studie)	⊕ ⊕ ⊕⊝MODERATE^1^	RR 1.40 (1.16 to 1.68)	Study population	
				491 per 1000	197 more per 1000 (from 79 more to 334 more)
				Moderate	
				491 per 1000	196 more per 1000 (from 79 more to 334 more)
Hematologic Adverse Events	1586 (3 studies)	⊕ ⊕ ⊕⊝MODERATE	RR 1.00 (0.86 to 1.16)	Study population	
				308HAE per 1000	0 fewer per 1000 (from 43 fewer to 49 more)
				Moderate	
				326 HAE per 1000	0 fewer per 1000 (from 46 fewer to 52 more)
Nonhematologic Adverse Events	1586 (3 studies)	⊕ ⊕ ⊕⊝MODERATE	RR 1.24 (1.02 to 1.52)	Study population	
				175 NHAE per 1000	42 more per 1000 (from 3 more to 91 more)
				Moderate	
				147 NHAE per 1000	35 more per 1000 (from 3 more to 76 more)
curative intent	1426 (2 studies)	⊕ ⊕ ⊕⊝MODERATE	RR 1.47 (1.55 to 1.88)	Study population	
				124 per 1000	59 more per 1000 (from 19 more to 110 more)
				Moderate	
				117 per 1000	59 more per 1000 (from 19 more to 110 more)
Overall survival	2576 (5 studies)	⊕ ⊕ ⊕⊝MODERATE	HR 0.89 (0.81 to 0.98)		
Progression free survival	2576 (5 studies)	⊕ ⊕ ⊕⊝MODERATE	HR 0.97 (0.90 to 1.05)		

*The basis for the assumed risk (e.g. the median control group risk across studies) is provided in footnotes. The corresponding risk (and its 95% confidence interval) is based on the assumed risk in the comparison group and the relative effect of the intervention (and its 95% CI). *CI:* Confidence interval; *RR:* Risk ratio;

GRADE Working Group grades of evidence High quality: Further research is very unlikely to change our confidence in the estimate of effect

Moderate quality: Further research is likely to have an important impact on our confidence in the estimate of effect and may change the estimate

Low quality: Further research is very likely to have an important impact on our confidence in the estimate of effect and is likely to change the estimate

Very low quality: We are very uncertain about the estimate

### Secondary outcomes

**Fig. 3:**
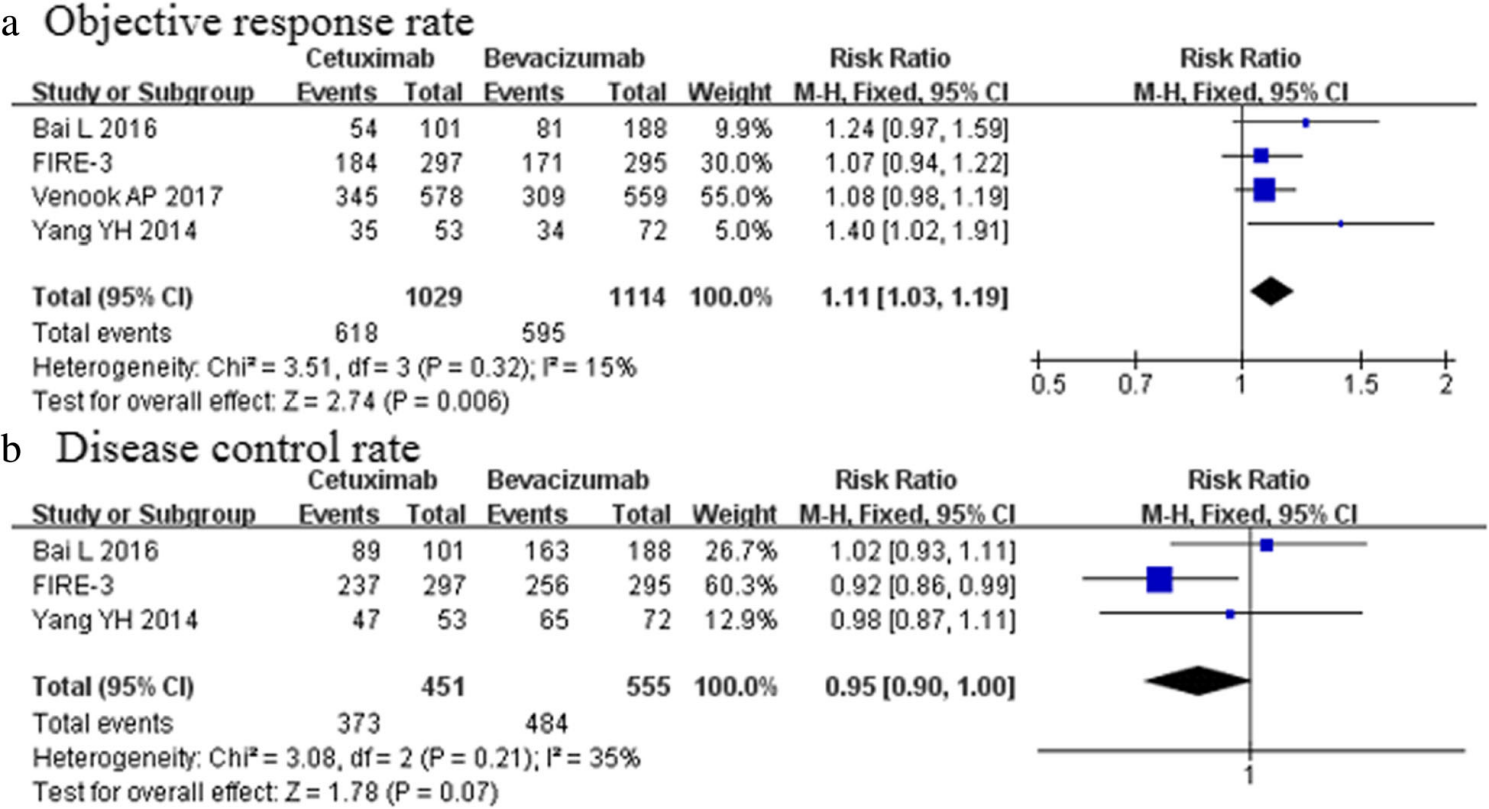
Objective response rate and disease control rate (**a** The meta-analysis documented a clear advantage of the cetuximab group over bevacizumab group in the ORR; **b** No significant differences in the DCR were noticed between the two groups)

**Fig. 4:**
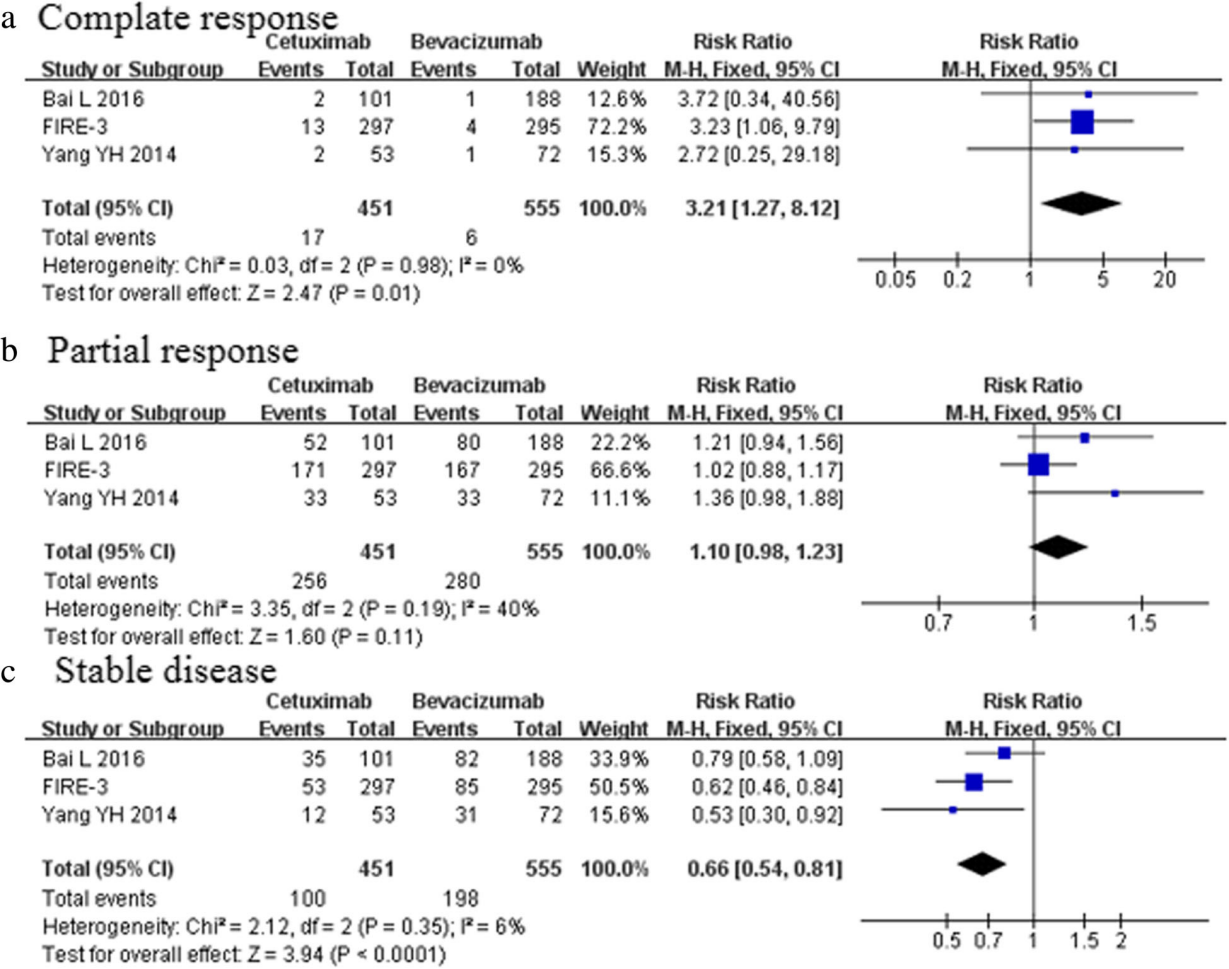
CR, PR and SD (**a** The meta-analysis of four trials showed a higher complete response in the cetuximab than bevacizumab group). **b** The partial response was not significantly different in both groups. 4c: bevacizumab group was associated with more stable disease)

The ORR was reported in four studies [5, 6, 24, 25]. The ORR was provided for 618 (60.1%) of 1029 patients who received cetuximab treatment and in 595 (53.4%) of 1114patients who received bevacizumab treatment. The meta-analysis documented a clear advantage of the cetuximab group over bevacizumab group [RR 1.11, 95%CI (1.03–1.19); *p* = 0.006, Fig. 3a, Table 3]. However, no significant differences in the DCR were noticed between the two groups [RR 0.95, 95%CI (0.90–1.00); *p* = 0.07, Fig. 3b, Table 4]. The meta-analysis of three trials [5, 24, 25] (*n* = 1006) showed a higher complete response in the cetuximab than bevacizumab group [RR 3.21, 95%CI (1.27–8.12); *p* = 0.01, Fig. 4a, Table 4].

**Fig. 5:**
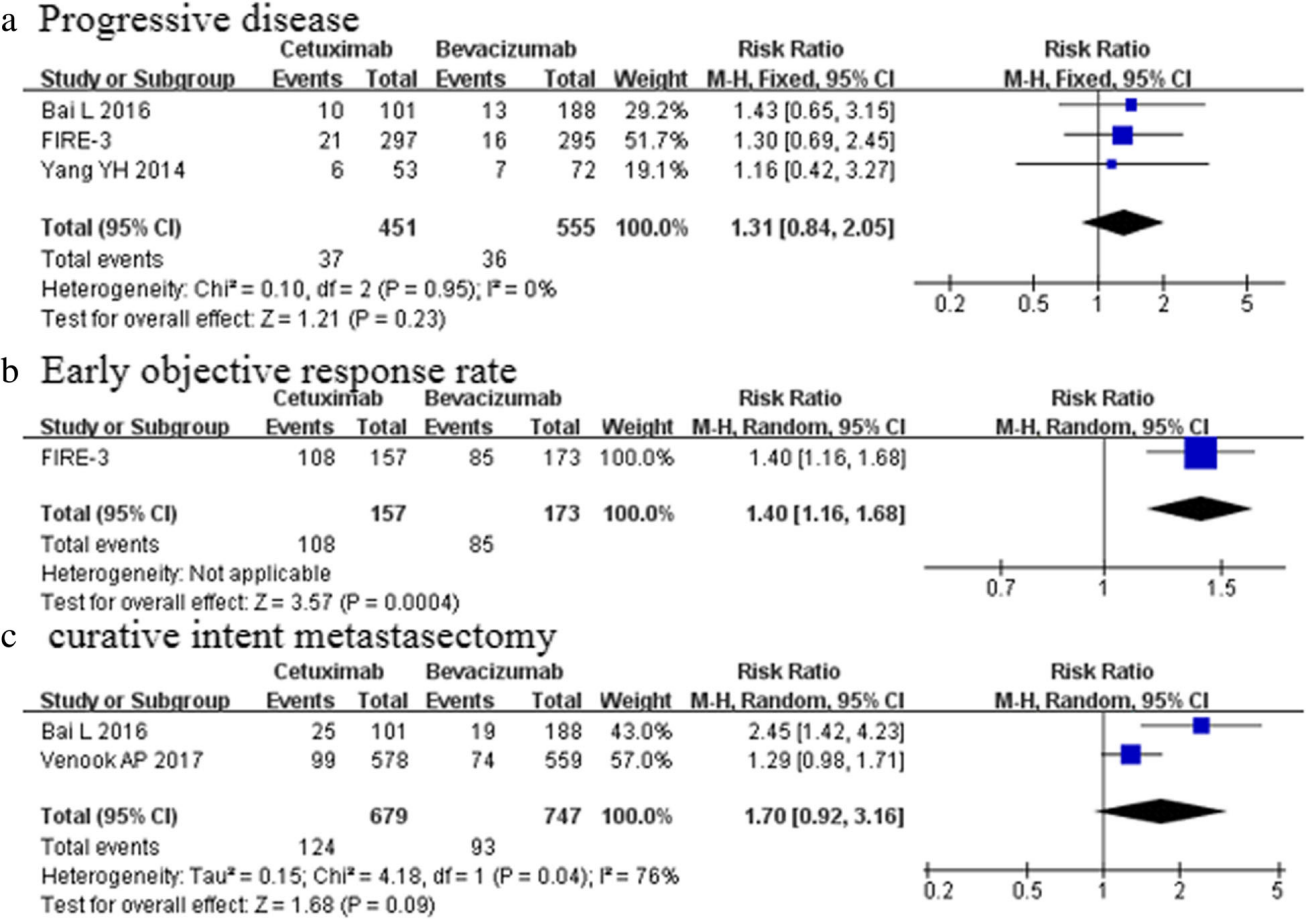
PD, EORR and CI (No significant difference was observed in progressive disease (Fig. 5**a**) and the rate of curative intent metastasectomy(Fig. 5**c**). Only FIRE-3 trial reported that Cetuximab-based regimens were associated with a relatively higher EORR compared with bevacizumab-based regimens(Fig. 5**b**)

Data were available for three of the included studies [5, 24, 25], involving 1006 patients. The partial response, pooled from the results of these studies, was not significantly different in both groups (56.8 vs 50.5%; *p* = 0.11, Fig. 4b, Table 4). However, bevacizumab group was associated with more stable disease (22.2 vs 35.7%; *p* < 0.0001, Fig. 4c). No significant difference was observed between the cetuximab group and bevacizumab group for progressive disease (8.2 vs 6.5%; *p* = 0.23, Fig. 5a) and the rate of curative intent metastasectomy (18.3 vs 12.5%; *p* = 0.09, Fig. 5c) (Table 4). Only FIRE-3 trials reported EORR and the depth of response. Cetuximab-based regimens were associated with a relatively higher EORR compared with bevacizumab-based regimens (68.8% vs 49.1%, *p* = 0.0004, Fig. 5b). FIRE-3 [24] documented a significantly greater median depth of response in the FOLFIRI plus cetuximab group relative to the FOLFIRI plus bevacizumab regimen(− 48.9% vs − 32.3%; *p* < 0.0001).

**Fig. 6:**
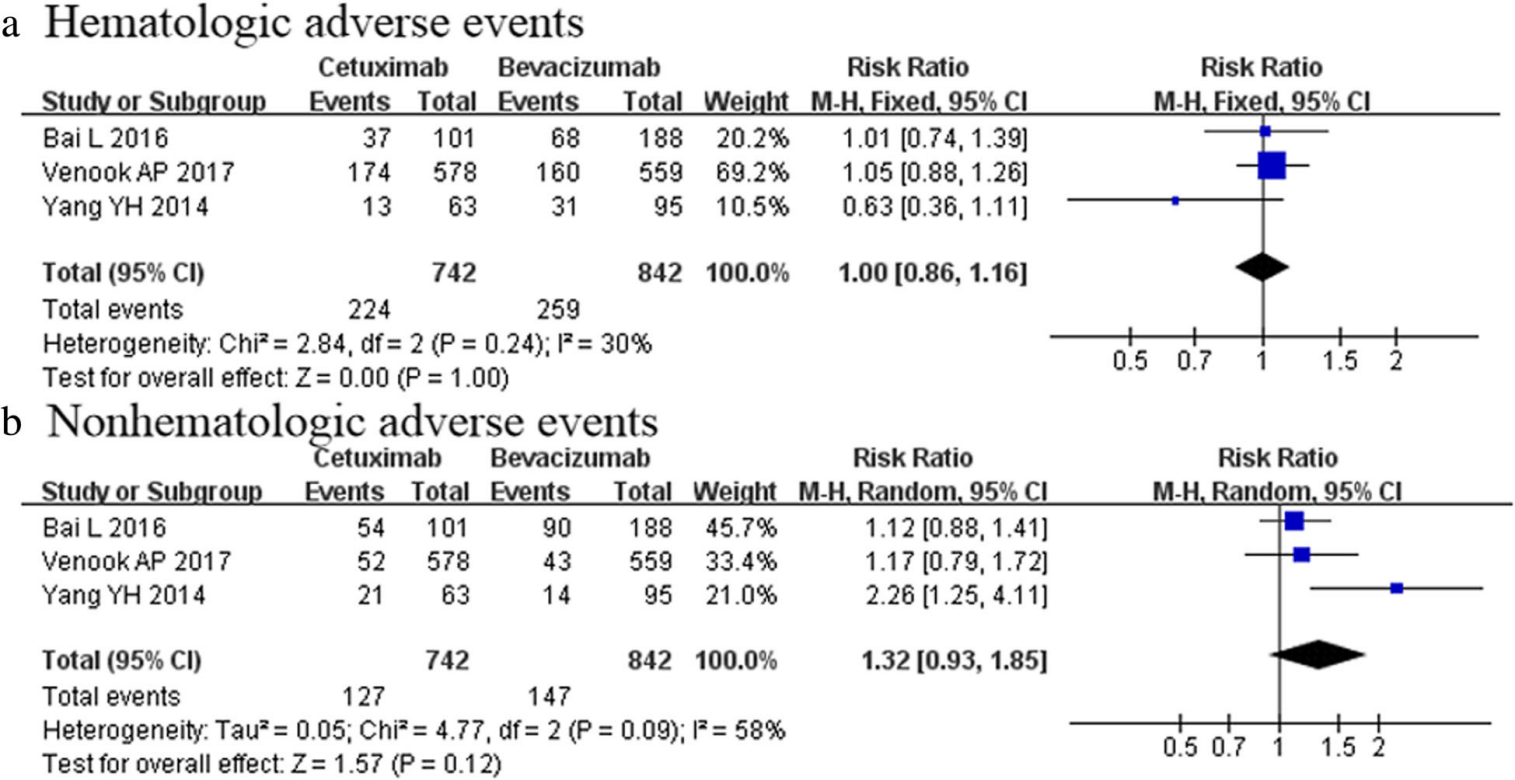
Adverse events (The risk of adverse events was comparable between the two groups (Fig. 6 **a** and **b**)

Three studies [6, 24, 25] with 1586 patients mentioned the incidence of grade 3 or higher adverse events. The risk of both hematologic adverse events and nonhematologic adverse events were comparable between the two groups [RR 1.00, 95%CI (0.85–1.16); *p* = 1.00, Fig. 6a; RR 1.32, 95%CI (0.93–1.85); *p* = 0.12, Fig. 6b, repectively]. (Table 4).

### Subgroup analyses

The inconsistent outcomes compared to the main analysis were found in ORR, DCR and curative intent metastasectomy. All RCTs reported ORR, and the subgroup meta-analysis showed that ORR was comparable between the two groups [RR 0.77, 95%CI (0.99–1.17); *p* = 0.07, Table 5]. Only one RCT reported DCR and curative intent metastasectomy, and The inconsistent outcomes compared to the main analysis were found. The subgroup meta-analysis revealed similar results between the two groups for the remaining outcomes (Table 5).

**Table 5:** Outcomes for meta analysis of RCTs

Outcomes	study	Number for patients		Heterogeneity(I^2^)	RR/HR	95%CI	*P* Value
		Cetuximab	bevacizumab				
Objective response rate	2 RCTs	529	480	0%	1.08	0.99–1.17	0.07
Disease control rate	1 RCT	237	256	–	0.92	0.86–0.99	0.02
Complete response	1 RCT	297	295	–	3.23	1.06–9.79	0.04
Partial response	1 RCT	171	167	–	1.02	0.88–1.17	0.81
Stable disease	1 RCT	297	295	–	0.62	0.46–0.84	0.002
Progressive disease	1 RCT	297	295	–	1.30	0.69–2.45	0.41
Early objective response rate	1 RCT	157	173	–	1.40	1.16–1.68	0.0004
Hematologic Adverse Events	1 RCT	578	559	–	1.05	0.88–1.26	0.58
Nonhematologic Adverse Events	1 RCT	578	559	–	1.17	0.79–1.72	0.43
Curative intent metastasectomy	1 RCT	578	559	–	1.29	0.98–1.71	0.07
Overall survival	2 RCTs	875	854	20%	0.86	0.76–0.96	0.007
Progression free survival	2 RCTs	875	854	19%	0.97	0.88–1.06	0.44

## Discussion

In the present meta-analysis of cetuximab versus bevacizumab as first-line therapy for patients with *RAS* and *BRAF* wild-type mCRC, we observed a significantly longer median OS in mCRC patients who were treated with cetuximab compared with those who received bevacizumab treatment. Furthermore, cetuximab was associated with higher ORR, lower stable disease, higher complete response and a greater median depth of response. However, no significant difference was observed between cetuximab and bevacizumab groups for PFS, DCR, partial response, progressive disease, curative intent metastasectomy, EORR and incidence of grade 3 or higher adverse events. In the subgroup meta-analyses of the RCTs, inconsistent results compared to the main analysis, however, were found, in the ORR, DCR and curative intent metastasectomy. To our knowledge, this is the first meta-analysis report of differences in the efficacy and safety of cetuximab as first-line therapy compared with bevacizumab for *RAS* and *BRAF* wild-type mCRC.

In patients with *RAS* wild-type mCRC, no preferable first-line biologic treatment has been clearly illustrated. A large randomized phase III trial demonstrated that EGFR (cetuximab and panitumumab) was not associated with clinical effectiveness in mCRC patients with *RAS* mutations [26]. A trial compared panitumumab to bevacizumab (PEAK), which included 156 patients with RAS WT/BRAF WT mCRC, aimed to assess the PFS of panitumumab versus bevacizumab in these patients [27]. Significantly longer median PFS was found in panitumumab group compared to bevacizumab group in the RAS wild-type (12.8 vs 10.1 months; HR = 0.68; *p* = 0.029) and RAS wild-type/BRAF wild-type (13.1 vs 10.1 months; HR = 0.61, *p* = 0.0075) mCRC. No significant difference was observed between cetuximab and bevacizumab groups in terms of OS.

There are two potential reasons why the advantages of DFS are not extended to OS. The first reason is that the numbers of patient with BRAF MT mCRC were different (panitumumab arm *n* = 11; bevacizumab arm *n* = 3), while the second reason is that the PEAK research was focused on PFS but not powered to detect any OS increase. However, the present systematic review included five trials, in which the median OS ranged from 28.3 to 37.8 months in the cetuximab group, being significantly higher than that in the bevacizumab group. There was no significant difference in PFS, and the PFS results of the meta-analysis are highly consistent with those reported in previous RCTs [5, 6]. Hence, large-scale randomized controlled studies are further needed to demonstrate this advantage in cetuximab-based regimens for *RAS* wild-type mCRC.

The University Hospital Dresden team demonstrated that liver metastasectomy rate is strongly correlated with the response rates [28]. In patients with mCRC, classic chemotherapy plus targeted agents evidenced significant improvement in the ORR and, consequently, increased the number of patients eligible for surgical treatment [29–32]. A recent systematic review and pooled analysis, designed to assess the clinical efficacy of FOLFOXIRI plus bevacizumab, demonstrated that classic chemotherapy combined with bevacizumab is associated with an improvement in ORR (69%) [33]. However, there is no difference in the rate of overall surgical conversions and rate of R0 surgical conversions in mCRC patients who received chemotherapy plus bevacizumab compared to chemotherapy. Our meta-analysis showed that cetuximab-based treatment is associated with a higher ORR in the cetuximab group over bevacizumab group (RR 1.09, *p* = 0.01). Moreover, it confirmed evidence of the present analysis, showing a significantly higher complete response in the cetuximab group relative to the bevacizumab group [RR 2.74, p = 0.01).

A trial noted that there were no significant differences in the incidence of adverse events of any grade and grade 3–4[27]. However, a systematic review and meta-analysis published in 2018 concluded that compared to a panitumumab-based scheme, cetuximab has a similar burden of toxicity regarding the rate of severe adverse events [34]. The present meta-analysis demonstrated that cetuximab significantly increased the rate of nonhematologic adverse events [RR 1.41, *p* = 0.02]. However, subgroup analysis of RCTs revealed that nonhematologic adverse events were comparable between cetuximab and bevacizumab treatment. Hematologic adverse events in the overall analysis and subgroup analysis are consistent. Therefore, the role of cetuximab warrants further discussion.

### Limitations

Our present meta-analysis has several limitations. First, significant heterogeneity was observed in stable disease, EORR, nonhematologic adverse events and curative intent. This disparity may be related to the differences in basic chemotherapy regimens. However, the National Comprehensive Cancer Network (NCCN) Clinical Practice Guidelines similarly recommend fluoropyrimidine in various schedules and combinations as first-line mainstay chemotherapy [35]. Second, primary tumor location is different between the two groups, and this may have a potential impact on the outcomes. A recent systematic review illustrated that patients with left-sided CRC might benefit more from anti-EGFR therapy than right-sided CRC patients [36]. However, further clinical trials, stratified for cancer location, are needed. Finally, different types of studies were included in the present survey. Although confounding factors were increased, the subgroup meta-analysis of RCTs was performed to verify the outcomes, and the current research still provides reliable evidence for clinicians.

## Conclusions

In conclusion, the current evidence indicates that compared to bevacizumab treatment, cetuximab provides a clinically relevant effect in the first-line treatment of *RAS* and *BRAF* wild-type mCRC on OS, ORR and complete response, at the cost of lower stable disease. Future research is still required to investigate the role of cetuximab on PFS, ORR and adverse events.

## Abbreviations

5-FU: 5-fluorouracil
ASCO: American Society of Clinical Oncology
B: Bevacizumab
C: Cetuximab
Co: Colon
CPCI-S: Web of Knowledge Conference Proceedings Citation Index-Science
DCR: Disease control rate
EGFR: Epidermal growth factor receptor
EORR: Early objective response rate;RECIST:Response Evaluation Criteria in Solid Tumors
ESMO: European Society for Medical Oncology
FOLFIRI: 5-FU, folinate and irinotecan
FOLFOX: 5-FU, OXA and LV
GRADE: Grading of Recommendations Assessment Development and Evaluation
L: Left
LV: Leucovorin
M: Multiple
mCRC: Metastatic colorectal cancer
mFOLFOX6: Modified 5-FU, LV and OXA
NOS: Newcastle–Ottawa Scale
OCSs: Observational cohort studies
ORR: Objective response rate
OS: Overall survival
OXA: Oxaliplatin
PFS: Progression-free survival
RCTs: Randomized controlled trials
Re: Rectum
Ri: Right
RR: Relative risks
SM: Synchronous Metastases
T: Transverse
TN: Total number
Un: Unknown
VEGF: Anti-vascular endothelial growth factor
XELOX: Capecitabine plus OXA
